# Assessment of Physiological Signals from Photoplethysmography Sensors Compared to an Electrocardiogram Sensor: A Validation Study in Daily Life

**DOI:** 10.3390/s24216826

**Published:** 2024-10-24

**Authors:** Rana Zia Ur Rehman, Meenakshi Chatterjee, Nikolay V. Manyakov, Melina Daans, Amanda Jackson, Andrea O’Brisky, Tacie Telesky, Sophie Smets, Pieter-Jan Berghmans, Dongyan Yang, Elena Reynoso, Molly V. Lucas, Yanran Huo, Vasanth T. Thirugnanam, Tommaso Mansi, Mark Morris

**Affiliations:** 1 Janssen Research & Development, Buckinghamshire HP12 4EG, UK; 2 Janssen Research & Development, Cambridge, MA 02142, USA; 3 Janssen Research & Development, 2340 Beerse, Belgium; 4 Janssen Research & Development, LLC, San Diego, CA 92121, USA; 5 Janssen Research & Development, Raritan, NJ 08869, USA; 6 Janssen Research & Development, Spring House, PA 19477, USA; 7 Janssen Research & Development, Titusville, NJ 08560, USA; 8 Janssen Research & Development, Brisbane, CA 94005, USA

**Keywords:** wearables, ECG, PPG, heart rate, heart rate variability, pulse rate, pulse rate variability, autonomic nervous system, remote monitoring, beat detection, multi-scale peak and trough detection algorithm

## Abstract

Wearables with photoplethysmography (PPG) sensors are being increasingly used in clinical research as a non-invasive, inexpensive method for remote monitoring of physiological health. Ensuring the accuracy and reliability of PPG-derived measurements is critical, as inaccuracies can impact research findings and clinical decisions. This paper systematically compares heart rate (HR) and heart rate variability (HRV) measures from PPG against an electrocardiogram (ECG) monitor in free-living settings. Two devices with PPG and one device with an ECG sensor were worn by 25 healthy volunteers for 10 days. PPG-derived HR and HRV showed reasonable accuracy and reliability, particularly during sleep, with mean absolute error < 1 beat for HR and 6–15 ms for HRV. The relative error of HRV estimated from PPG varied with activity type and was higher than during the resting state by 14–51%. The accuracy of HR/HRV was impacted by the proportion of usable data, body posture, and epoch length. The multi-scale peak and trough detection algorithm demonstrated superior performance in detecting beats from PPG signals, with an F1 score of 89% during sleep. The study demonstrates the trade-offs of utilizing PPG measurements for remote monitoring in daily life and identifies optimal use conditions by recommending enhancements.

## 1. Introduction

The continuous assessment of heart rate (HR) and heart rate variability (HRV) in daily life is crucial for pre-emptive health monitoring and management of chronic diseases [1,2]. Diseases such as inflammatory bowel disease, including Crohn’s disease and ulcerative colitis, are linked to complex interactions between the autonomic nervous system and gut inflammation, with stress exacerbating the condition [3]. HRV, as a reliable indicator of autonomic nervous system balance, can reflect physical and emotional stress and is predictive of cardiovascular morbidity and mortality [4]. Continuous HR monitoring assists in detecting arrhythmias and other heart conditions that may go unnoticed in episodic clinical tests [5,6]. Thus, daily life variability in HR and HRV can provide a more accurate picture of an individual’s health. Wearable PPG based devices are increasingly being used in continuous monitoring of HR and HRV. PPG devices use optical sensors to detect blood volume changes in tissue and are convenient for a variety of settings, including personal health applications. The PPG sensors, however, do not technically measure HR but rather specifically measure the pulse rate (PR) from the blood volume change. Therefore, they do not measure HRV, but they do technically measure pulse rate variability (PRV). However, for the purpose of simplicity, we will use the terms HR and HRV for both ECG- and PPG-derived measurements but specifically note if they have been derived from a PPG or ECG sensor. Various studies [7,8,9] have validated the utility of PPG in different contexts, including resting, post-exercise, and field conditions, demonstrating its versatility and effectiveness.

Despite their promise, the utilization of PPG-based devices in clinical research presents several limitations that must be first understood and addressed before deploying them in clinical trials. A key issue is the impact of individual differences, such as skin tone, age, and gender, on PPG readings [10,11,12,13,14]. Physiological aspects like respiration, venous pulsation, and body temperature can introduce noise in PPG signals [15,16,17,18]. Additionally, external factors such as motion artifacts, ambient light, and pressure applied to the skin can affect the accuracy of PPG devices [19,20,21,22,23]. Since this can negatively impact the quality of the data, careful consideration is necessary in the selection and use of PPG devices for health monitoring.

PPG-based devices can provide a plethora of features primarily categorized into HR and HRV. HRV features can be further categorized into time domain, frequency domain, and non-linear domain features [4]. The previous studies validating physiological measures from PPG devices have been primarily conducted in controlled environments, which do not represent the challenges encountered in data quality, compliance, and reliability when used in free-living settings. Furthermore, the results reporting accuracy of PPG-derived HRV vary in literature, and there is a paucity of studies evaluating HRV features in free-living conditions. For example, Polar H10 reported good agreement with an ECG-based device for interbeat intervals (R-R intervals) and HR; however, results were not reported for any HRV feature [24]. Polar V800 showed weak absolute agreement (intra class correlation (ICC) < 0.3) with an ECG-based device for time domain HRV features such as root mean square of successive differences (RMSSD) and standard deviation of normal RR (NN) intervals (SDNN) [25]. In another study [26], six wearable devices were evaluated in sleep lab settings, where good agreement was found for HR and poor agreement for RMSSD. The validation of PPG-derived R-R intervals and HR was performed in [27], where analysis was conducted under a resting state and over a very short recording time of 45 s. Another study validated the Samsung smartwatch during awake and asleep state against an ECG-based device using an epoch length of 5-min, showing weak to moderate correlation for HR and HRV during awake state and moderate to strong correlation during asleep state [28].

For precise heartbeat detection, especially under varying cardiac conditions, it is crucial to collect and analyze raw PPG data [29]. Innovative algorithms play a pivotal role in this context. For example, a study employed a peak detection algorithm for smartwatch PPG signals, resulting in significantly enhanced heart rate estimation accuracy in scenarios including atrial fibrillation [30]. A bidirectional recurrent denoising auto-encoder method demonstrated effectiveness in denoising and accentuating PPG waveform features, thereby improving signal quality and heart rate detection [31]. Additionally, the implementation of a novel hybrid motion artifact detection-reduction method using support vector machines has been shown to improve the accuracy of motion artifact detection, which is crucial for real-time vital sign monitoring [32]. However, before application of such complex algorithms, there is a need to first understand the baseline performance of traditional algorithms [33].

In this study, we address the gap in the existing research by performing a rigorous validation of PPG-derived physiological measures. Specifically, the objectives are as follows:

(1) Assessment of the feasibility of collecting continuous data from PPG devices and their usability in daily life settings.
(2) Validation of the HR and HRV derived from PPG devices during awake, asleep, or the full day period compared to that of an ECG sensor.
(3) Investigation of impact of data quality, body posture, activity types, epoch length for HRV estimation, use of dominant vs. non-dominant hand on estimation of HR and HRV from PPG devices.
(4) Assessment of the test–retest reliability of PPG-derived HR and HRV features in the daily life settings under awake, asleep, and full day periods.
(5) Investigation of the performance of seven algorithms to detect beats from the raw PPG waveform signal to identify potentially superior approaches to analyze noisy sensor data in the daily life.

## 2. Methods

### 2.1. Study Participants

Twenty-five healthy volunteers participated in this non-interventional exploratory study. The clinical study was performed at a single clinical pharmacology unit in Belgium during November 2022 to March 2023. The individuals were 18 years of age or older and determined to be healthy based on physical examination, medical history, and vital signs recorded during screening. The participants were required to comply with study instructions: wear two PPG devices and an ECG sensor simultaneously for two consecutive five-day periods and complete daily morning questionnaires and an end-of-study survey. The participants were excluded from the study if they had current or prior medical conditions, concomitant therapies, and current or prior participation in a clinical study within 28 days of the start of this study. Furthermore, they were also excluded if they had any constraints on sleep schedule, exposure to high frequency equipment during monitoring period, or tattoos on their wrist or torso potentially interfering with PPG/ECG measurements. They were not allowed to perform intensive exercise nor activities submerging devices in water during the monitoring period. The study received approval from the ethics committee of UZA/UAntwerp (3738-BUN B3002022000126). All participants provided written informed consent. This study followed the procedure according to the Declaration of Helsinki.

### 2.2. Measurement Setup

Two PPG-based devices (the Whoop 4.0 [34] and the Corsano CardioWatch 287-1B [35]) and one ECG device (Vital Patch [36]) were used in this study. The Corsano CardioWatch 287-1B (manufacturer: Corsano Healthcare BV, Den Haag, The Netherlands) is a wrist-worn research-based home monitoring device and consists of an accelerometer, PPG sensors, and a battery. The bracelet connects via Bluetooth to a mobile app and then to Corsano’s secure cloud. It sampled acceleration and PPG signals at 25 Hz and used firmware version 4.13. In addition the to raw data, it also provides the following readings: heart rate, R-R intervals, heart rate variability (e.g., RMSSD), respiration rate, activity count, activity type, steps, energy expenditure, and sleep stages.

The Whoop 4.0 (manufacturer: Whoop, Boston, MA, USA) is a wrist worn commercial device and captures continuous data from its accelerometer and PPG sensors. The Whoop strap containing the actual measuring device connects via Bluetooth to a mobile app and then to secure cloud storage. The firmware version 41.9.2-11.5 was used for Whoop. The device measures the following: sleep duration, sleep staging, sleep disturbances, sleep efficiency, resting heart rate, heart rate variability (RMSSD), respiratory rate, SpO2, heart rate, R-R intervals, and skin temperature.

The Vital Patch device (manufacturer: VitalConnect Inc, San Jose, CA, USA) is adhered to the chest and provides high quality single-lead ECG readings of heart rate and heart rate variability. The VitalConnect device wirelessly transmits data from the Vital Patch sensor to a smartphone and then to the PhysIQ (manufacturer: PhysIQ, Chicago, IL, USA) cloud for storage and analysis [2]. The firmware version used for PhysIQ was 3.5.1.4. The patch is equipped with ECG and accelerometer sensors to measure various physiological parameters such as heart rate, R-R intervals, respiratory rate, body temperature, skin temperature, fall detection, activity (including step count), posture (body position relative to gravity), and sleep stages.

### 2.3. Study Design

**Figure 1:**
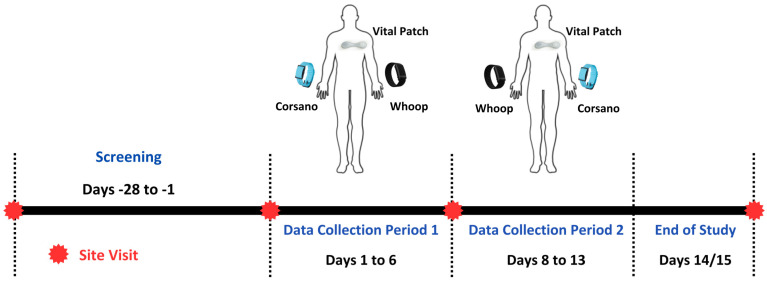
Study design, device attachments, and continuous data collection periods.

This study included two periods of data collection (Figure 1) from daily life for passive home-based remote monitoring. The first data collection period included Day 1 through Day 6. The second data collection period included Day 8 through Day 13. On Day 1, the participants began wearing all three devices (Whoop 4, Corsano Cardiowatch 287-1B, Vital Patch) simultaneously. During the first data collection period, the Vital Patch was worn on the chest. The Whoop 4 was worn on the participant’s non-dominant hand, while the Corsano Cardiowatch 287-1B was worn on their dominant hand. The devices were worn for 5 consecutive days and nights, which included at least 1 weekend night. On Day 8, the participants began the second data collection period wearing all three devices. The Vital Patch was worn in the designated location on the chest as indicated during the site visit. The Corsano Cardiowatch 287-1B was worn on the participant’s non-dominant hand, while the Whoop 4 was worn on their dominant hand. The devices were worn for an additional 5 consecutive days and nights, which included at least 1 weekend night. During the whole data collection period, on every third day participants were instructed to charge the devices for at least three hours in the evening.

Participants completed a daily morning questionnaire, prompted at 9 a.m., which asked two questions: the time at which the participant went to bed the previous night and the time at which the participant woke up that day. Responses from the morning questionnaire were collected on Day 2–Day 6 and Day 9–Day 13. A participant was considered to have completed the study if the participant had completed the two data collection periods of five consecutive days and nights, daily device assessments, and the end of study survey.

### 2.4. Device Usability Assessment

An end-of-study survey was used to evaluate the usability of each device in daily life. The following usability aspects of each device were assessed on a Likert scale from 1–5 (1 indicates strongly disagree to 5 indicates strongly agree) based on the modified version of the standardized questionnaire for the system usability scale [37].

(1) I thought it was easy putting the device on and taking it off
(2) I experienced discomfort wearing the device
(3) I experienced trouble sleeping due to the device
(4) My device stayed in place
(5) I would like to use the device frequently
(6) I found the device easy to use
(7) I needed support of a technical person to be able to use the device
(8) I experienced restrictions in my daily activities due to device
(9) I felt confident wearing the device
(10) I needed to learn a lot of things before I could get going with the device
(11) I found various functions of the device were well integrated (wearing, charging, application features, etc.)
(12) I found the device very cumbersome to use
(13) I experienced skin irritability wearing the device

## 3. Data Analysis

### 3.1. Coverage Assessment

To assess the feasibility of the PPG devices to be used for daily continuous monitoring, coverage of each PPG device data was calculated in two different ways. The first assessment focused on the collection of continuous raw PPG/Acceleration data in daily life, and the second assessment concentrated on the ability of the devices to be used for continuous beat detection in daily life. For the first assessment, the raw data coverage of each device was calculated on an hourly basis as a percentage of the available sample data points in a particular hour to the intended number of samples in this hour. Hourly coverage was further aggregated into full day (across 24 h) and different parts of the day (midnight to 8 a.m., 8 a.m. to 8 p.m., and 8 p.m. to midnight) for reporting. To assess the feasibility of the PPG devices for continuous beat detection in daily life, R-R intervals obtained from the devices were utilized. For simplicity, we use R-R intervals to refer to beat-to-beat intervals for PPG and R-R intervals for ECG. For coverage estimation, a 5-min epoch length was considered for the analysis. The data coverage within this epoch was calculated first, and if there were at least 40% of the data present, this epoch was considered valid. Further, the processed data hourly coverage was estimated by counting the valid epochs within an hour divided by the intended possible number of 5-min epochs in that hour. The hourly coverage was further aggregated into full-day periods (24 h) and specific time intervals for reporting: midnight to 8 a.m., 8 a.m. to 8 p.m., and 8 p.m. to midnight. The charging times of the devices were not adjusted in the coverage calculation to simulate real-world daily life scenarios.

### 3.2. PPG/ECG Device Data (R-R Intervals) Processing

From each device, valid 5-min epoch R-R interval data were further processed before feature engineering. The R-R interval data provided by each device were processed first by sorting it based on the timestamps and removing any duplicates. The R-R intervals were then cleaned by removing the outliers based on unrealistic physiological values and ectopic beats to extract the cleaned normal-to-normal (N-N) intervals for robust feature engineering [38]. The procedure for computing normal-to-normal (N-N) intervals from R-R intervals consisted of several sequential steps. Initially, R-R interval outliers, defined as the ones outside of the 300–2000 ms range [4,39], were identified and replaced with NaN values to clean the data. Subsequently, any NaN values in between the reliable R-R interval values were interpolated using a linear interpolation. This step ensures continuity in the data by filling gaps with interpolated values. Following this, ectopic beats, or abnormal heartbeats, were removed from the interpolated R-R intervals using the Malik method (where the consecutive interval deviation is more than 20% from the previous one) [40]. This generated a series of N-N intervals representing the time intervals between consecutive normal heartbeats. However, the ectopic beat removal may introduce new NaN values, necessitating a second interpolation step. The same interpolation method applied earlier was utilized again to fill in any remaining NaN values within the N-N intervals. Due to the validation nature of this work in daily life, the same interpolation technique was used for all features instead of considering different interpolation techniques for each HRV feature [41]. The result is a list of interpolated N-N intervals, where physiological unrealistic and ectopic beats have been systematically removed, and missing values have been filled in. This comprehensive pre-processing approach ensures a robust and adaptable foundation for further heart rate variability (HRV) analysis. However, an ablation study was also conducted to compare the impact of the current interpolation technique with that of no interpolation of HRV features (Appendix B).

### 3.3. Feature Engineering

Cleaned epochs of 5-min N-N intervals from each device were further used to extract HRV features related to time, frequency, and non-linear domains along with the mean value of heart rate and N-N intervals. For the further validation analysis, only representative features from each domain were considered and described in Table 1. More information regarding feature definitions can be found in the work by Shaffer and Ginsberg [4].

### 3.4. Factors Affecting the PPG Device Performance

In addition to measuring HR and HRV throughout the full day (from midnight to next midnight for each day), it is crucial to consider the influence of the body’s circadian rhythm. This natural rhythm can cause HR/HRV features to vary between day and night, subsequently affecting their accuracy. Particularly during periods of sleep with minimal wrist movement, HRV features tend to be more accurate compared to wakeful periods when daily activities are performed. Morning questionnaire responses were used to crop the data based on subjective asleep and awake timings for each day.

Moreover, there are several other factors, including data coverage within epochs used for HRV estimation, postural transitions, activity types, walking vs. non-walking, epoch length, and device position, which can impact the estimation of PPG derived HR/HRV features.

*Coverage within a 5-min epoch used for HRV estimation:* Continuous detection of beats from PPG raw data without gaps is key for reliable HRV feature calculation. The impact of R-R data coverage within 5-min epochs was investigated by increasing the coverage threshold from 40% to 100% with increments of 10%.

*Postural transitions:* Body posture in daily life can also impact the PPG data reliability. Postural information obtained from Vital Patch, such as upright, reclined, lying right, lying left, prone, and supine information, was used to label each 5-min epoch of data used for HR/HRV estimation. A specific posture label was assigned based on its dominance within the 5-min epoch of data in case the participant changed a posture with this 5-min time interval.

*Activity type:* Performance of the PPG devices was also assessed under various daily living activities such as cycling, rest, walking, and running provided by the Corsano device after processing the accelerometer data.

*Walking* vs. *non-walking:* Specifically, walking detected by the chest-worn device (Vital Patch), which can be more reliable compared to wrist-worn devices, was also used to check the performance of the PPG devices.

*Epoch length:* The impact of epoch length on the error rate of HRV estimation during asleep, awake, and full day periods was explored by using epoch lengths of 10, 30, and 60 min. Apart from these epoch lengths, whole asleep and awake periods were also investigated.

*Dominant* vs. *non-dominant hand:* Five complete days of data from each collection period from each subject were used to investigate the impact of wearing the PPG devices on dominant vs. non-dominant hands during the asleep, awake, and full day periods.

### 3.5. Data Consideration for Reliability Assessment of HRV Features

**Figure 2:**
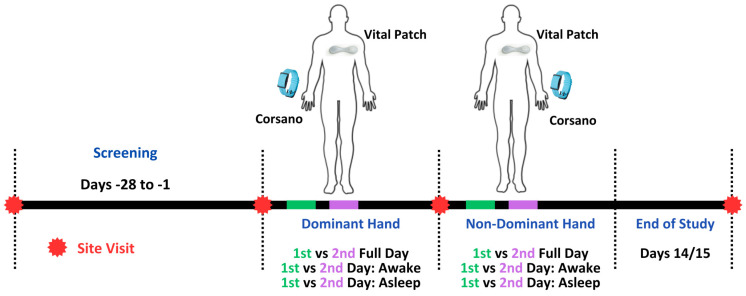
Data consideration for the reliability assessment of the HR and HRV features.

Reliability of the HRV features was further explored. For reliability assessment, as shown in Figure 2, the data were considered separately when the device was attached to the dominant and non-dominant hand. Within each period of device attachment, two separate full days (24 h) were considered. To compute reliability, the spearman correlation was performed between HRV estimates obtained from synchronized 5-min epochs between day 1 and day 2. Similarly, a reliability assessment was performed during the first day awake/asleep period with the second day awake/asleep period based on the synchronized 5-min epochs of the HRV features.

### 3.6. Algorithms for Beat Detection from Raw PPG and ECG Data

Only Corsano provided raw PPG data at 25 Hz frequency. Seven open-source algorithms, which performed well on PPG data in a previous study [33], were employed to detect beats from the raw PPG data. A short description of each algorithm is provided in Table 2. The methodology for beat detection from raw PPG data methodology was adopted from the prior work [42]. Briefly, raw PPG green signals were subjected to band-pass filtration to remove extraneous cardiac frequencies. Beats were identified over specific length PPG intervals with certain overlap. Redundant detections from overlaps were excluded. Segments with a continuous flat signal exceeding 0.2 s, often due to sensor disengagement or saturation, were discarded. For validation, the beat (R peaks) detected from the simultaneously recorded ECG signal by two different beat detectors were used as reference following the previous work [33]. The two beat detectors utilized were the ‘jqrs’, which employ the Pan and Tompkins technique [43,44], and Clifford’s ‘rpeakdetect’ ECG beat detector [42]. Outputs from these two algorithms were aligned and then merged, with ‘correct’ beats in the merged signal being those identified by both within a 150 ms interval. Any 20-s segments without consensus between the two detectors were omitted from the analysis.

The alignment between PPG and ECG detected beats is not always exact. Therefore, we used the methodology proposed by Charlton et al. [33]. Briefly, to synchronize the PPG beats with ECG, the time discrepancy between each ECG beat and its nearest PPG counterpart was computed. If this difference was less than 150 ms, the beat was deemed accurately identified. In increments of 20 ms for shifting either PPG or ECG beat sequence, this alignment procedure was repeated while offsetting the beats by lags ranging from −10 to 10 s. The offset yielding the most accurate beat identifications was taken as the genuine lag and utilized to harmonize beat timings.

### 3.7. Validation Approach and Statistical Analysis

**Figure 3:**
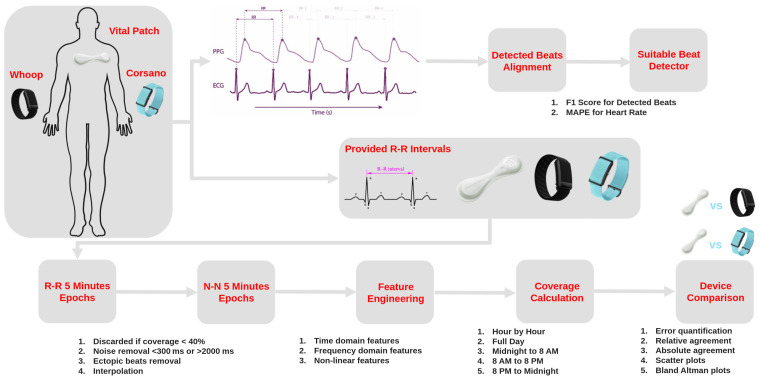
Validation workflow of wearable PPG-based devices for HR and HRV.

The validation workflow is shown in Figure 3, where the performance of the devices was assessed based on the provided R-R intervals. Each PPG device feature extracted from N-N intervals was compared with the ECG-derived features during the same time interval. All the devices were synchronized based on local UTC time. During this validation analysis, depending on the coverage and pre-processing of the R-R intervals, HRV features from various domains were calculated (Table 1). To assess the accuracy of measurements, the relative error and absolute error were quantified. The relative agreement between HRV features of the PPG device and ECG was assessed with correlation coefficient. Absolute agreement between the devices was calculated with the ICC coefficient. The relationship between the PPG and ECG features was further visualized through the scatter plots. In addition, to analyze the difference between devices (PPG vs. ECG), Bland–Altman plots were used, and other validation metrics such as bias (mean error) and 95% limit of agreements were calculated. The reliability of the HRV features was assessed with the ICC coefficient within each data collection period for the dominant and non-dominant hands during asleep, awake, and full day periods. The feasibility of continuous remote data collection in home settings was assessed by an evaluation of the coverage and usability of devices. Average values of the coverage and usability along with the standard deviation were reported as bar plots.

The performance of the beat detectors on the raw PPG data was assessed by comparing the detected beats with the reference ECG beats. A tolerance window of ±150 ms as described in Section 3.6 was used for assessing the correctness of beat detection between PPG and ECG. For example, if the detected beat from the PPG data is present within this window of the reference ECG beat, then it is considered to be correctly identified. For full day, asleep, and awake periods, the numbers of correct beats, reference beats, and PPG beats were identified to calculate the sensitivity and positive predictive value (PPV). The harmonic mean of PPV and sensitivity, as well as the F1 score, was used to identify the best performing beat detectors. Furthermore, for time points corresponding to each beat, the HR was using the preceding 8 s interval [33]. The performance of HR estimation for different beat detectors was assessed as mean absolute percentage error (MAPE). All the performance metrics for the evaluation of beat detectors are reported as median values along with 95% confidence intervals.

A mathematical formulation of the evaluation metrics is given below.

**Mean Absolute Error (MAE):** MAE is the average of the absolute differences between measured (PPG) and true values (ECG), calculated as

$$MAE=\frac{1}{n}\sum_{i=1}^{n}\left|x_{measurement,\;i}-x_{true,i}\right|$$

where

- $x_{measurement,i}$ is the i-th measured value of the epoch,
- $x_{true,i}$ is the *i*-th true value of the epoch,
- n is the total number of measurements.

**Mean Relative Error (MRE):** MRE measures the average relative error as a percentage of the true value:

$$MRE=\frac{1}{n}\sum_{i=1}^{n}\frac{\left|x_{measurement,\;i}-x_{true,i}\right|}{x_{true,i}}\times 100$$

**Spearman Correlation:** This correlation coefficient (ρ) assesses the rank-order relationship between two variables, which is non-parametric and useful when there is a non-linear relationship between the variables:

$$\rho =1-\frac{6\;\sum d_{i}^{2}}{n(n^{2}-1)}$$

where

- $d_{i}$ is the difference between the ranks of $x_{measurement,i}$ and $x_{true,i}$
- n is the total number of measurements.

**Intra-Class Correlation (ICC):** ICC 2,1 (two-way random effects, single measurement) quantifies the degree of agreement between two sets of measurements, considering both individual variability and systematic differences:

$$ICC\left(2,1\right)=\frac{MSR-MSE}{MSR+\left(k-1\right)MSE+\frac{k(MSC-MSR)}{n}}$$

where

- MSR is the mean square of rows (subjects)
- MSE is the mean square error (residual)
- MSC is the mean square for columns (devices)
- k is the number of devices
- n is the number of subjects

**Sensitivity:** The proportion of true positives (TP) correctly identified by the algorithm to TP and FN (false negative):

$$Sensitivity=\frac{TP}{TP+FN}$$

**Positive Predictive Value (PPV):** The proportion of predicted positives that are true positives to TP and FP (false positive):

$$PPV=\frac{TP}{TP+FP}$$

**F1 Score:** The F1 score is the harmonic mean of precision (PPV) and sensitivity:

$$F1=2\times \frac{PPV\times Sensitivity}{PPV+Sensitivity}$$

## 4. Results

The demographic characteristics for the participating subjects collected at screening are shown in Table 3. The average age of participants was 46.9 years, with majority (n = 17) being female (F). Participants had a body mass index in the range of 24.68 ± 3.10 kg/m^2^.

### 4.1. Feasibility of PPG Devices in Daily Life

The feasibility analysis is divided into (1) the coverage analysis of the raw PPG and the R-R interval data processed by the device and (2) the usability analysis of the PPG device deployment in daily life.

#### 4.1.1. A Coverage Analysis

**Figure 4:**
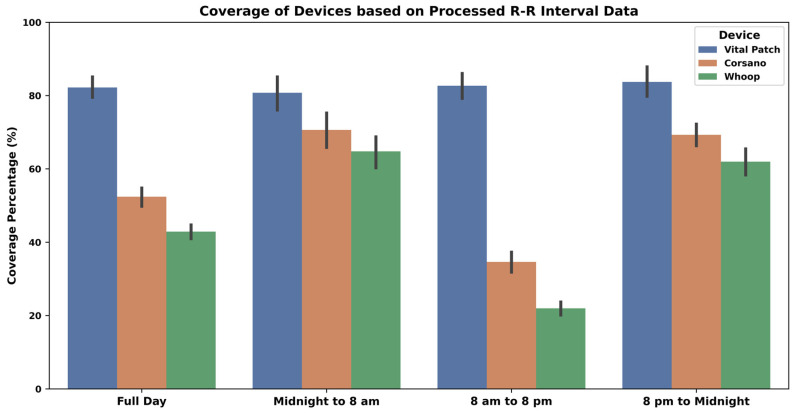
R-R interval data coverage—where the height of the bars indicates the average coverage values while the whiskers correspond to 95% confidence intervals.

The coverage for raw ECG/PPG/Acceleration data and processed R-R interval data is presented as median value along with min and max values in Table 4. Additionally, the coverage of the processed R-R intervals is also shown in Figure 4 as average values along with a 95% confidence interval. Vital Patch provided 100% coverage of the raw ECG data during most of the study days whenever it was attached to the body. Corsano had similar coverage for the raw PPG data. However, the Whoop device had slightly less coverage each day when compared to Corsano.

As demonstrated in Figure 4 and Table 4, it is clear that the ECG-based device detected more beats in the data and had better coverage than PPG devices. PPG devices detected fewer beats during the daytime as compared to night. Therefore, the median coverage varied from 44–52% during a full day period to 77–88% only during the night. Overall, Corsano has better coverage for the processed R-R interval data as compared to Whoop.

#### 4.1.2. Usability Analysis

**Figure 5:**
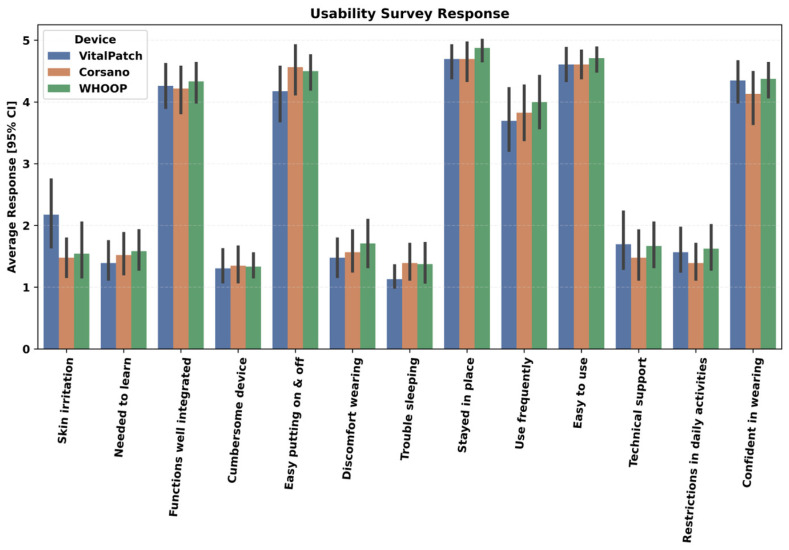
Usability evaluation of devices. Participants completed an end-of-study survey comprising a 13-item questionnaire assessing the usability of each device. Figure here shows abbreviated versions of the questions described in Section 2.4.

The second aspect of the feasibility assessment was to investigate the usability of the wearables. A 13-item questionnaire (Section 2.4) was answered by each participant at the end of the study, and their responses are shown in Figure 5. The results indicated that all devices were easy to use, they stayed in place, had low discomfort when wearing, were not cumbersome, and functioned well. However, for Whoop, participants indicated a slight need to learn more before one could get going with the device and a need for more technical support when compared to Corsano. Furthermore, Vital Patch had higher skin irritation, followed by Whoop and then Corsano.

### 4.2. Mutual Data for Validation

**Figure 6:**
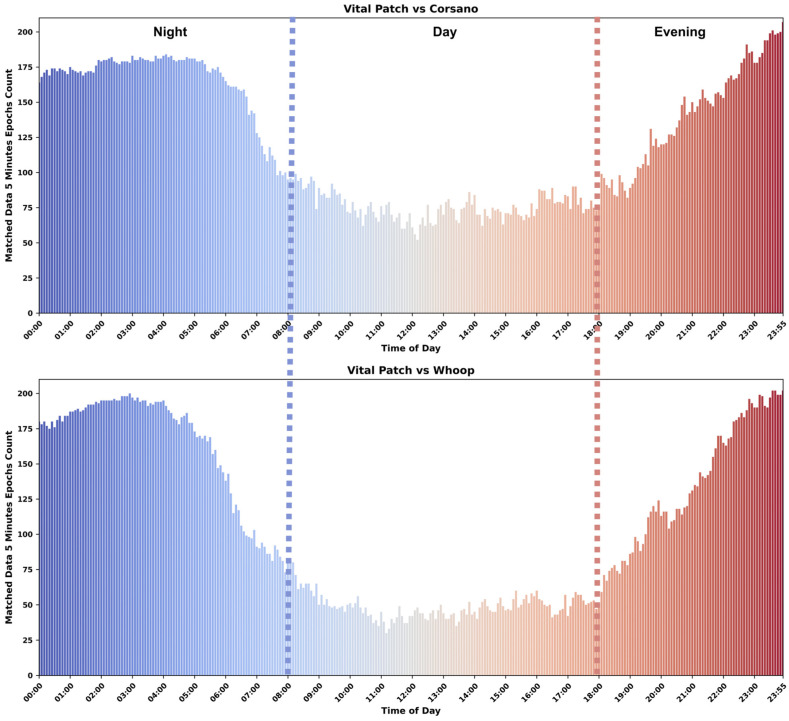
Bar plot showing the availability of 5-min epochs, which are valid for both devices, across days and participants.

Based on coverage analysis in Section 4.1.1, Vital Patch had more processed R-R interval data each day when compared to the Corsano and Whoop. Therefore, it is critical to understand when the majority of the data are available for the validation. Since validation could be performed only using data from such epochs, which are considered valid for both devices under comparison, we computed the number of such mutually valid epochs for different times a day for Corsano–Vital Patch and Whoop–Vital Patch pairs. Figure 6 shows the comparison for both Corsano and Whoop with Vital Patch, where each bar corresponds to the matched number of valid 5-min epochs between an ECG- and PPG-based device across all participants and days. Figure 6 is further divided into the night, day, and evening. During night and evening, both devices had more matched data when compared to daytime for the validation.

### 4.3. Performance of the PPG Devices

**Figure 7:**
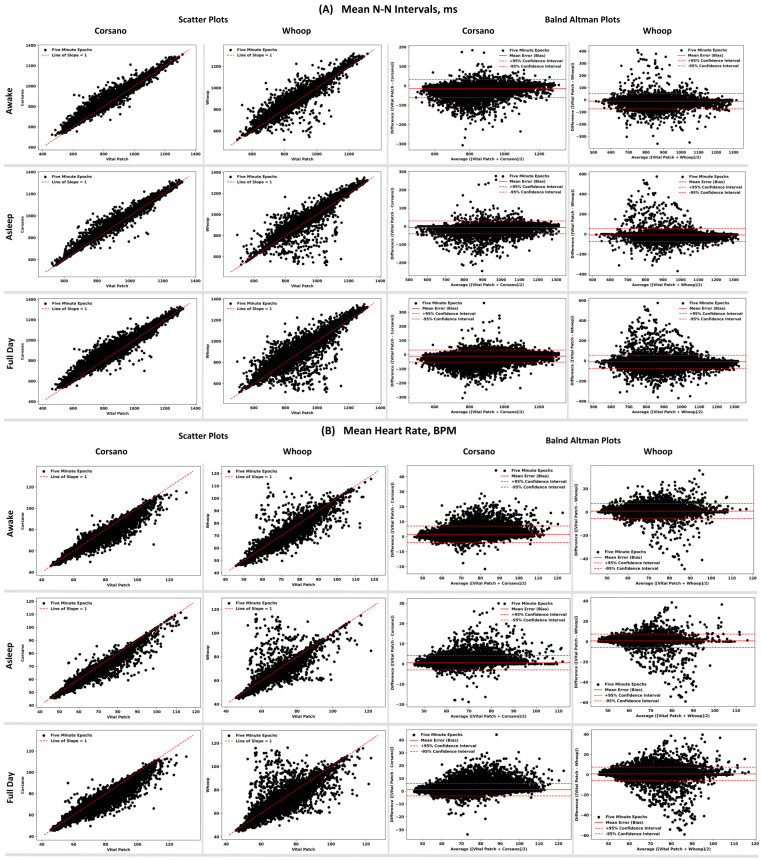
Scatter and Bland–Altman plots showing the performance of the PPG devices compared to ECG device for (**A**) mean N-N intervals (top) and (**B**) HR (bottom) across 5-min epochs.

The results of the comparison of HR/HRV features obtained from PPG devices to the same features derived from ECG device are shown in Table 5. The performance comparison is reported based on three time intervals: when participants were asleep, awake, and during the full day. Scatter plots and Bland–Altman plots showing alignment between the N-N interval and heart rate are shown in Figure 7.

During the full day period, Whoop had higher error in N-N intervals and HR compared to Corsano. However, both PPG devices had good relative and absolute agreement in N-N intervals and HR with the ECG device. For time domain HRV features like RMSSD, and SDNN, both devices performed similarly in terms of their agreements with ECG. In the frequency domain, both devices had a high error rate during the full day and low absolute agreement.

The error rate during asleep time was lower than awake time for all HR/HRV features. The mean error for HR during asleep time was less than one beat and for N-N intervals was less than 10 ms. However, both devices overestimated the N-N intervals during both awake and asleep periods when compared to the ECG device. Time domain HRV during asleep time had errors in a range of 6–10 ms for Corsano, while Whoop had errors in a range of 7–15 ms. For frequency domain features, the error rate was drastically lower during the asleep time compared to awake time for both devices, the reduction being more in the case of Corsano than Whoop. Both PPG devices had a lower error rate and good agreement with the ECG device during asleep time as compared to awake time.

### 4.4. Factors Impacting the Performance of a PPG-Based Device

The impact of coverage within epoch, body posture, daily life activities, epoch length, dominant vs. non-dominant hands, on the performance of the PPG-based device was explored only for the Corsano device, which provided the raw PPG data. While exploring the impact of these factors, a relative error in percentage is reported for the representative HR/HRV features.

**Figure 8:**
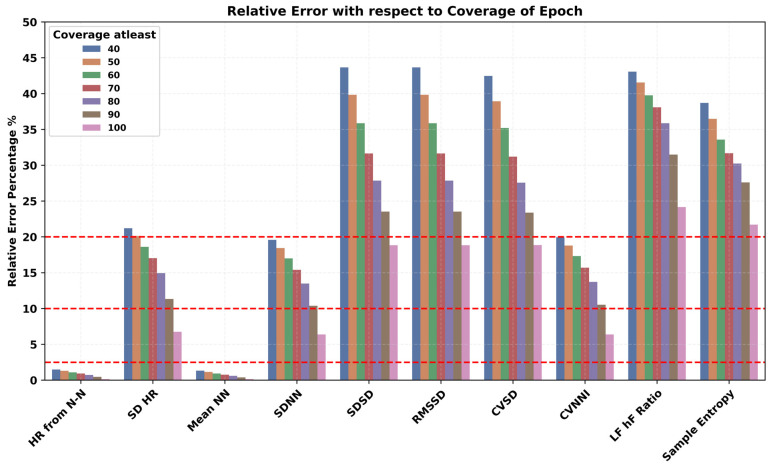
Impact of coverage within a 5-min epoch on the accuracy of PPG-derived HR and HRV features.

We hypothesized that increasing the threshold for quantifying a valid epoch will lead to a more accurate estimation of HR/HRV features. Therefore, we experimented with the coverage threshold for a 5-min epoch from 40% to 100% and investigated its impact on the performance of HR/HRV features as shown in Figure 8. The relative error decreased for all the HR/HRV features on increasing the coverage. The error rate for RMSSD and SDNN reduced by approximately around 20% and 10%, respectively. Similar trends were observed for frequency and non-linear domain HRV features.

**Figure 9:**
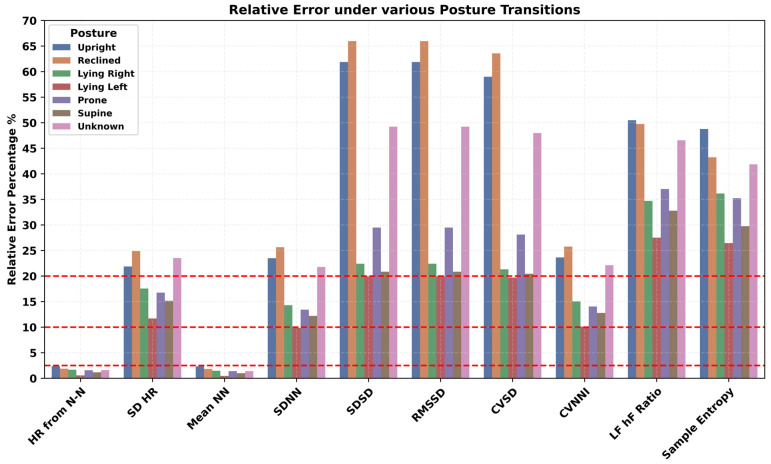
Impact of body posture on accuracy of PPG-derived HR and HRV features.

**Figure 10:**
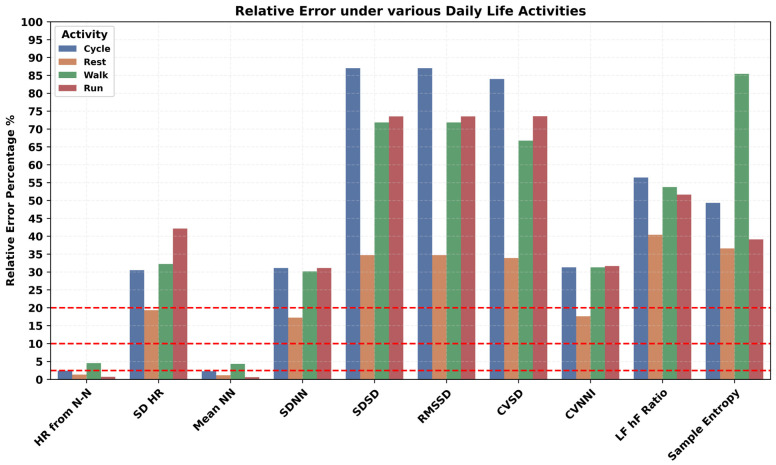
Impact of daily life activities on accuracy of PPG-derived HR and HRV features.

**Figure 11:**
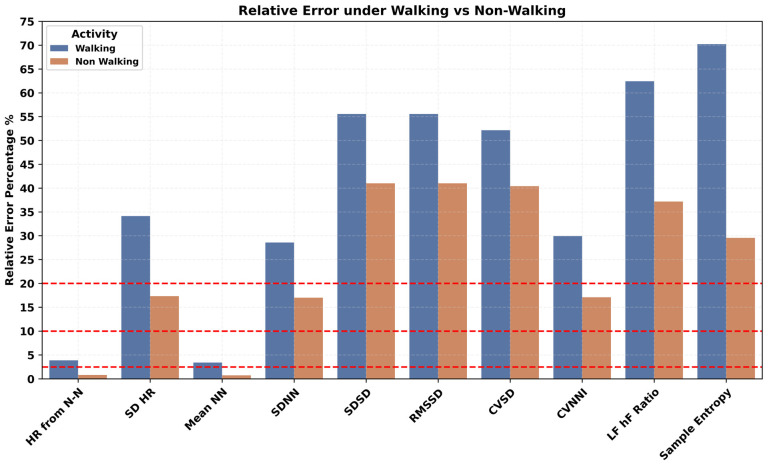
Impact of mobility on accuracy of PPG-derived HR and HRV features.

The impact of a variety of body postures, such as upright, reclined, lying left and right, prone, and supine positions, on PPG-derived HR/HRV features was investigated and presented in Figure 9. In all HR/HRV features, a higher error was observed during the upright and reclined positions. Specific lying positions played a critical role, such as lying face down in the prone position, which had a higher error rate compared to the supine position for the HR/HRV features. Similarly, lying on the right side has higher error than lying on the left side. The most appropriate position for PPG HRV features engineering was the lying position and especially lying on the left side. Various repetitive and cyclic daily living activities such as cycling, walking, and running resulted in a higher error rate in all the HR/HRV features, as shown in Figure 10. The lowest relative error was observed during rest, where the SDNN has a relatively lower error than RMSSD. Mobility, here, walking vs. non-walking, influenced the accuracy of PPG features, as shown in Figure 11. During walking, the relative error was higher than non-walking for all the HR/HRV features. The difference in the relative error between the two activities was 10% for SDNN and 15% for RMSSD. This difference increased further for the frequency and non-linear features as shown in Figure 11. Wearing the device on the dominant or non-dominant hand did not exhibit any significant difference (Appendix A).

**Figure 12:**
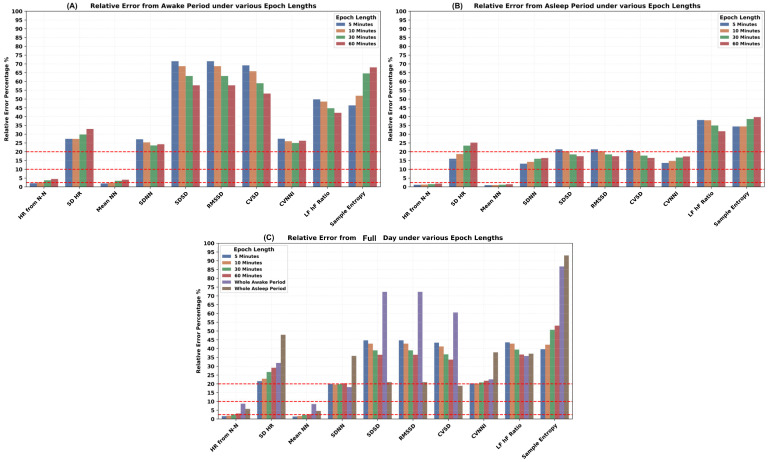
Impact of epoch length on accuracy of PPG-derived HR and HRV features.

The impact of epoch length was explored under the awake (Figure 12A), asleep (Figure 12B), and full day periods (Figure 12C). Various epoch lengths, such as the default 5 min, 10 min, 30 min, and 60 min, whole awake time during the day, and whole asleep time during the night for all HR and HRV features estimation, were investigated. Interestingly, the correspondence of different PPG-derived HR and HRV features to ECG-derived ones behaves differently under various epoch lengths. The relative error rate increased for the mean HR and mean N-N intervals under both asleep and awake conditions while increasing the epoch length from 5 to 60 min. In contrast, for the majority of the time domain HRV features, the error rate reduced while increasing the epoch lengths. However, the relative error increased for the SDNN during the asleep period and did not follow the same trend as during the awake period. Similarly, the frequency domain HRV features also resulted in a lower error rate while increasing the epoch length. For non-linear features such as the sample entropy, the relative error went up with the increase in the epoch length. The asleep period resulted in the lowest relative error for the RMSSD. Again, SDNN behaved differently than RMSSD, where the SDNN performed well during the awake period compared to the asleep period.

### 4.5. Test–Retest Reliability of the HR and HRV Features in Daily Life

**Figure 13:**
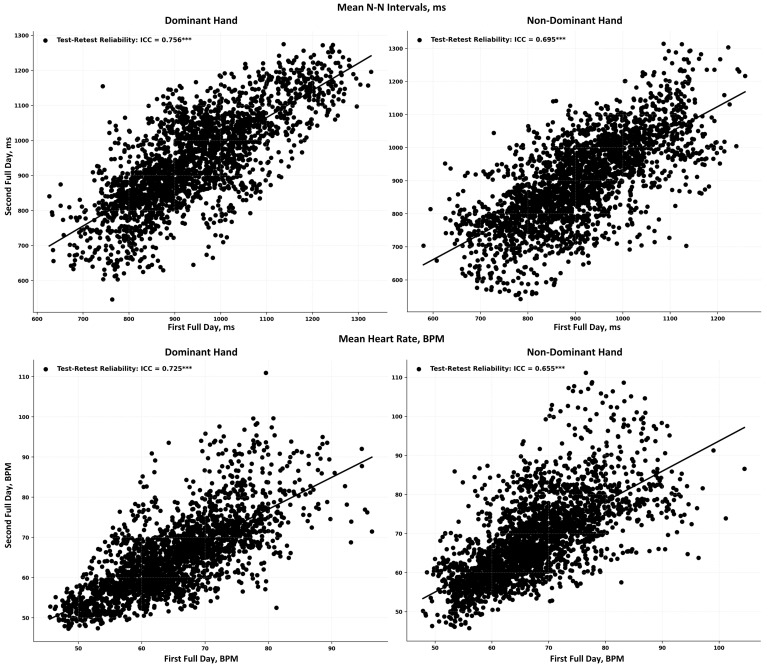
Test–retest reliability of mean N-N intervals (top row) and HR (bottom row) from dominant (left column) and non-dominant (right column) hands. Each black dot represents a synchronized 5-min epoch. *** indicates *p*-value is <0.001.

The test–retest reliability of the HRV features obtained from Corsano is shown in Table 6. Scatter plots for alignment of N-N intervals and HR estimated at the same time at two different adjacent days are provided in Figure 13 for both dominant and non-dominant hands. The dominant hand had higher reliability than non-dominant hands under awake, asleep, and full day periods. For all time domain HRV features, test–retest reliability was higher during the asleep periods compared to the awake periods. Frequency domain features also showed higher reliability during the asleep period compared to the awake period, except the LF/HF ratio, which had higher reliability during the awake period. Among all features, RMSSD from time domain HRV and HF from the frequency domain HRV had better reliability.

### 4.6. Performance of Beat Detection Algorithms on the Noisy PPG Sensor Data

**Figure 14:**
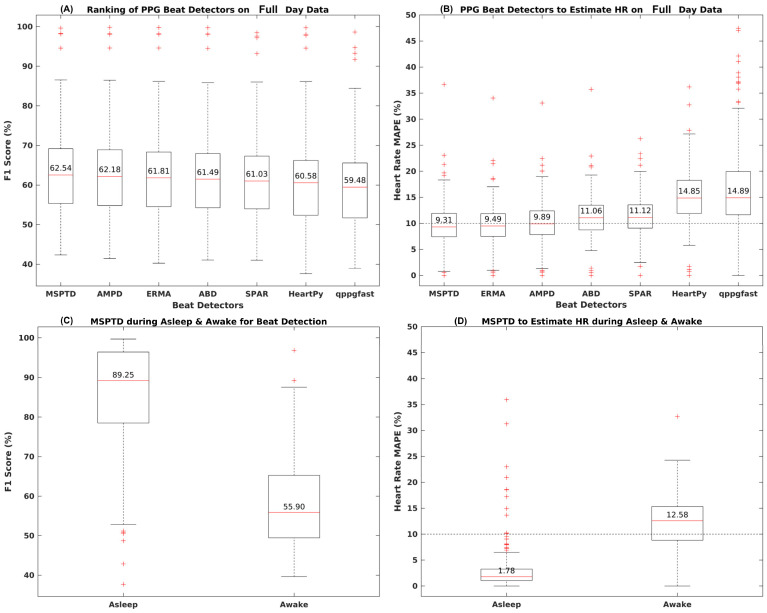
Performance of various beat detectors on the noisy PPG data compared to R-peak detected from ECG in daily life recordings.

A suite of seven beat detection algorithms was investigated to evaluate their performance in analyzing raw PPG signals collected in daily life settings. The performance of each algorithm to detect beat and mean absolute percentage error (MAPE) for HR estimation is shown in Figure 14A,B and Table 7. Considering all the recorded data, the performance of the algorithms for the beat detection assessed via F1 score appeared to be similar (Figure 14A). When the performance was evaluated using MAPE for HR estimation, differences between algorithm performance emerged, with only three algorithms, MSPDT, ERMA, and AMPD, having MAPE lower than 10%. The beat detection F1 score performance of these algorithms was around 62% with a 95% CI in the range of 44% to 87%. The median sensitivity was 59–61% with a 95% CI in the range of 42% to 95%. The positive predictive value (PPV) was 63–64%. For HR estimation, apart from MAPE, the mean absolute error (MAE) for three top-performing algorithms was 7 BPM with a negative bias of around 2–3 beats. For the interbeat interval estimation, the MAE error for the top performing algorithms ranged in between 249 ms and 337 ms (Table 7).

The top-performing algorithm, MSPDT, which is a modified version of the AMPD algorithm, was explored further on a subset of data corresponding to the asleep and awake periods separately. The results are shown in Figure 14C,D and Table 8. The F1 score for beat detection with MSPTD went up from 55% during the awake period to 89% during the asleep period. The sensitivity of the MSPTD during asleep was 89% and 54% during the awake period. Furthermore, the PPV was also 89% during the asleep and 57% during the awake period (Figure 14C). The MAPE for HR estimation significantly reduced from 12.58% during the awake period to 1.78% during the asleep period (Figure 14D). The MAE for HR estimation was around 1 beat during asleep and 10 beats during the awake period. Additionally, MSPTD MAE for the interbeat intervals was also significantly reduced to 54 ms during the asleep period compared to 220 ms during the awake period.

## 5. Discussion

In this paper, we performed a systematic validation of physiological measures derived from PPG devices collected over multiple days in free living settings, which, to the best of our knowledge, is the first of its kind. Specifically, we investigated the feasibility of the remote collection of physiological measures from PPG devices in daily life, the usability of such devices, and the accuracy of derived features at different time intervals of day: awake, asleep, or throughout the day. We examined the impact of body posture, mobility, and data coverage on the accuracy of the features and evaluated their test–retest reliability. Furthermore, we quantified the performance of various algorithms to detect heartbeats from noisy raw PPG signals.

### 5.1. Feasibility of PPG Data Collection and Device Performance

Our results showed that users found PPG devices comfortable and easy to use, resulting in positive usability ratings. While the ECG data yielded 100% coverage for most days and consistent beat detection throughout the day, PPG devices showed variability in coverage and detected fewer beats at daytime compared to nighttime. The prior work has shown coverage rates ranging from 70% to 90% for estimating heart rate and 50% to 90% for estimating pulse arrival time (PAT) or pulse amplitude variability (PAV), with variations based on sensor location and quality [53].

Corsano generally had a lower error in estimating N-N intervals and HR compared to Whoop. Both devices showed good relative and absolute agreement with ECG-derived features and performed similarly for time-domain HRV features like RMSSD and SDNN. In the frequency domain, both devices showed higher error rates and lower absolute agreement. This is in line with the previous work, which showed that the frequency-domain HRV features explored previously in elderly vascular patients, especially those associated with high-frequency content, were systematically overestimated [54]. This overestimation resulted in a relatively large bias, indicating that care should be taken in interpreting these parameters when derived from wrist-worn wearable devices. The error rates in our work were lower during asleep than awake periods for all HRV features. Corsano demonstrated superior performance, particularly during asleep periods, with mean errors for heart rate and N-N intervals being minimal. This finding from Corsano and Whoop aligns with the general observation that wearable PPG devices tend to perform better in situations with minimal motion, such as during sleep [28,55]. However, these conclusions cannot be generalized to all PPG devices without further comparative evaluations. Both devices, however, tended to overestimate N-N intervals across all conditions. This is due to the inherent limitations of PPG technology in accurately capturing beat-to-beat intervals under varying conditions. Motion artifacts, diverse skin types, and signal crossover, among others, could contribute to such potential inaccuracies in PPG-derived measurements [56].

### 5.2. Factors Impacting the Performance of PPG-Based Devices and Derived Features

The performance of PPG devices was influenced by several factors. The coverage within a 5-min epoch significantly impacted the accuracy of HR/HRV features. Increasing the epoch coverage from 40% to 100% decreased the relative error in HR/HRV features, with a 20% reduction in error rate for RMSSD and over 10% for SDNN. The estimation of SDNN showed small biases when compared with the ECG reference, while RMSSD exhibited systematic overestimation in the range of 10%. This indicates that the accuracy and reliability of HRV measurements from PPG can significantly vary based on the quality and coverage of the data [54].

Human body posture during daily life activities influenced the estimation of PPG-derived features. Higher error rates in HR/HRV features were observed in upright and reclined positions compared to specific lying positions. The prone position showed a higher error rate than the supine position. Lying on the right side resulted in higher errors than lying on the left side, irrespective of whether the device was on the right or left hand. The most suitable position for PPG HRV features engineering was the lying position, particularly on the left side. It is likely that during upright positions, there is more movement in the upper body and hands compared to lying positions. Daily life activities like cycling, walking, and running resulted in higher error rates in all PPG HR/HRV features. The difference in relative error between walking and non-walking was significant, nearly 10% for SDNN and 15% for RMSSD. This difference increased further for frequency and non-linear HRV features, suggesting that PPG data should ideally be recorded during non-walking activities for more accurate results. According to a prior study, absolute error across wearable devices was 30% higher on average during motion than during rest for HR/HRV [56]. Another study showed that wearable devices can detect heart rates accurately under resting conditions; however, daily life physical activities impact the performance of these PPG-based devices [57].

Different epoch lengths (5, 10, 30, 60 min) for HR and HRV feature estimation were analyzed. There was an increase in error for mean HR and N-N intervals with longer epochs and a decrease in error for most time domain HRV features. For frequency domain and non-linear features, error generally increased with longer epochs. The lowest error for RMSSD was noted during the asleep period. Short-term spectral HRV analysis, typically conducted over a few minutes, is useful for tracking rapid changes in cardiac autonomic function. In contrast, long-term spectral HRV analysis, ranging from an hour to a full day, provides a more stable assessment of autonomic function, capturing longer fluctuations and better predicting prognosis. However, long-term analyses are more resource-intensive and susceptible to noise and variability due to environmental factors and daily activities [58]. Furthermore, HRV indices vary significantly across distinct sleep epochs, challenging the practice of aggregating HRV indices across these epochs from the whole asleep period. The previous work [59] found that both rapid eye movement (REM) and non-REM stage 2 (N2) sleep epochs showed a change in HRV indices throughout the night. This variability suggests that aggregating HRV indices across sleep stages could obscure important transient effects.

### 5.3. Reliability of the PPG-Based Assessments in Daily Life

Different levels of reliability based on hand dominance and state of consciousness (awake/asleep) were observed in this study. Generally, the features extracted from a signal recorded from the dominant hand showed higher reliability across all periods. Time domain HRV features exhibited greater test–retest reliability during asleep periods than awake periods. Similarly, frequency domain HRV features, except the LF/HF ratio, showed better reliability during asleep periods. In contrast, the LF/HF ratio had improved reliability during awake periods. Non-linear HRV features displayed better reliability with the dominant hand during asleep periods. In the context of HRV reliability during repetitive low-intensity activities, a study found that the time interval between repeated measurements did not influence the HRV values, indicating HRV’s reliability under different low intensity activities [60]. Furthermore, the previous work [61] also showed HRV’s potential as a reliable measure in varying states of consciousness, supporting our observed findings of varying levels of HRV reliability based on the state of consciousness (awake/asleep).

### 5.4. Algorithms for Beat Detection from Noisy PPG Sensor Data During Daily Life

In the evaluation of algorithms for processing noisy PPG data, three out of seven algorithms—MSPDT, ERMA, and AMPD—stood out by achieving heart rate estimation accuracy with a MAPE below 10%. Furthermore, MSPDT showed significantly better performance during asleep than when awake, with improvements in beat detection and a substantial reduction in both MAPE and MAE for inter-beat intervals. In terms of algorithmic logic, the AMPD algorithm’s [46] strength lies in its local maxima scalogram matrix, which identifies optimal scales for capturing the most local maxima in a PPG signal. This scale-based approach allows for more precise beat detection amidst variable signal quality. ERMA [47] uses Butterworth bandpass filtering and applies specific moving averages to emphasize systolic peaks and individual beats. This method enhances the signal’s features relevant for accurate beat detection, even in the presence of noise. MSPDT [49] employs a modified version of the AMPD algorithm, optimizing the detection of local maxima and minima in PPG signals. This approach is particularly effective in differentiating true signal peaks from noise, which is crucial in noisy environments. The previous work also highlights the importance of choosing the right PPG beat detector algorithm, noting that algorithms like MSPDT show complementary performance characteristics in different conditions, such as rest and exercise, and in different patient demographics [33,62]. More details on the MSPDT algorithm and their implementation can be found in a prior work [63].

### 5.5. Key Insights and Recommendations

This study provides crucial insights into the use of PPG devices for HRV monitoring in daily life settings. It reveals that while PPG devices like the Corsano and Whoop show reasonable accuracy in comparison to ECG data, especially during sleep, their performance is affected by various factors such as data coverage, body posture, activity types, and epoch length. Time domain HRV features exhibit higher reliability during asleep periods. Frequency domain features, except for the LF/HF ratio, show better agreement during asleep periods. Additionally, algorithms like MSPDT, ERMA, and AMPD are effective in processing noisy PPG data, with MSPDT being particularly effective during asleep periods.

Based on these insights, several recommendations are proposed. There is a need for manufacturers to enhance data coverage and optimize algorithms to improve PPG device accuracy, particularly during daytime activities. Emphasis should be placed on design aspects like sensor placement and stability to minimize motion artifacts. Users and clinicians should be informed of the optimal conditions for PPG device use, understanding their limitations, especially during high-intensity activities. Future research should focus on reducing the impact of motion artifacts and other external factors on PPG data quality and developing more robust algorithms for various real-life conditions. Finally, while PPG devices offer a convenient means for HRV monitoring, caution is advised in interpreting data for clinical decisions, especially in scenarios where high precision is required. These recommendations highlight the potential of PPG devices in HRV monitoring while acknowledging the necessity for further improvements in technology and usage guidelines.

### 5.6. Study Limitations

The study presents several limitations. It focused on healthy individuals, limiting its assessment to the patient population. The scope of this work has been limited to two PPG devices, and future work should explore a broader range of PPG technologies, such as those worn on fingers. Future work should also include heterogenous demographics to assess the impact of skin tone, which has not been investigated in this work. In this study, the impact of charging on data coverage has not been explored, which may be investigated in future studies through the collection of self-reported questionnaires on participant’s charging times or duration throughout the study. Moreover, the use of signal-processing-based beat detectors, while reliable, highlights the necessity for the development of novel algorithms. These new algorithms would be instrumental in enhancing beat detection accuracy in challenging scenarios, marking a key direction for future advancements in wearable health technology.

## 6. Conclusions

This work evaluated the performance of wearable PPG devices for HR and HRV monitoring in daily life settings, thus enhancing the applicability of our findings to real-world scenarios, which is vital for both consumer and clinical applications. Our results showed that, overall, PPG-based devices showed promise in monitoring physiological features. The data coverage of PPG devices was lower during active daytime hours, and beat detection capability was noticeably diminished during the day. Data coverage and beat detection accuracy were high, especially when the users were sleeping. Agreement varied by coverage threshold, epoch length, body posture, and activity type. Users found the devices comfortable and user-friendly, resulting in good positive usability ratings. A MSPDT algorithm performed best in detecting beats from noisy raw PPG signals. The study recommends optimal PPG data collection strategy and analysis methodologies that should be employed in a clinical trial where such devices may be used for remote health monitoring to minimize estimation error of HR and HRV and thus aid in accurate clinical decision making.

## Figures and Tables

**Table 1:** List of HR and HRV features, along with their definitions, used in the validation analysis. HRV features were extracted from 5-min epochs of N-N intervals.

Feature (Units)	Domain	Definition
Mean HR (BPM)	Time	The average heart rate.
Std HR (BPM)	Time	Standard deviation of heart rate
Mean N-N (ms)	Time	The mean of the N-N intervals, which are the normal-to-normal intervals or the time between successive normal heartbeats.
SDNN (ms)	Time	The standard deviation of the N-N intervals, indicating overall HRV.
SDSD (ms)	Time	The standard deviation of successive differences between adjacent N-N intervals, emphasizing short-term variations.
RMSSD (ms)	Time	The square root of the mean of the sum of the squares of differences between adjacent N-N intervals.
CVSD	Time	Coefficient of variation of successive differences between adjacent N-N intervals.
CVNN	Time	Coefficient of variation equal to the ratio of SDNN divided by Mean N-N intervals
LF (ms^2^)	Frequency	Low-frequency power spectral density (0.04 to 0.15 Hz)
HF (ms^2^)	Frequency	High-frequency power spectral density (0.15 to 0.40 Hz)
LF/HF	Frequency	A ratio of LF to HF
Sample Entropy	Non-linear	A non-linear measure that quantifies the complexity or irregularity of the HRV signal

**Table 2:** Brief description of the beat detection algorithms used during validation analysis.

Algorithm Name	Description
1. Automatic Beat Detection (ABD) by [45]	This algorithm computes a Fourier-based power spectral density (PSD) to isolate the signal’s primary energy bands. Subsequently, the signal undergoes band-pass filtering, emphasizing distinct heart rate frequencies. This is complemented by derivative-based filtering, which makes rapid signal transitions prominent. A modification is made to the percentile threshold, initially set around the 90th percentile in the original algorithm but later modified to the 75th percentile, to detect peaks in the derivative (75th percentile used in this work). After filtering, the algorithm identifies pulse peaks. To enhance accuracy, it corrects potential peak location errors, removes false positives based on interbeat intervals and median heart rate thresholds, and integrates missing peaks to account for false negatives.
2. Automatic Multi-Scale Peak Detection (AMPD) by [46]	The PPG signal is first detrended and then segmented into overlapping windows of 6 s in duration with 20% overall. Within these windows, the algorithm constructs a local maxima scalogram (LMS) matrix. Rows of the LMS corresponded to scales, spanning from a single sample up to half of the window’s duration, while columns represent individual PPG samples. The algorithm updates specific LMS matrix entries to zero when a PPG sample surpasses its neighboring values at a given scale, indicating a local maximum. By analyzing the LMS, the algorithm determines the optimal scale (lambda), which represents the scale capturing the most local maxima. The LMS matrix is then truncated to retain only scales smaller than this optimal lambda. The final beat detection step identifies beats as those PPG samples that are recognized as local maxima across all the retained scales in the truncated LMS.
3. Event-Related Moving Averages (ERMA) by [47]	The algorithm processes the PPG signal with a Butterworth bandpass filter, limiting the frequency range to 0.5 Hz to 8 Hz. The filtered signal was subsequently squared, ensuring non-negative values. Two specific moving averages are then applied: the first, with a 111 ms duration, is designed to emphasize systolic peaks, while the second, spanning 667 ms, makes individual beats prominent. A threshold is computed as 2% of the squared signal’s mean. Within 111 ms windows, beats are pinpointed when the first moving average exceeds the sum of the second moving average and the defined threshold.
4. HeartPy by [48]	This algorithm starts by processing the PPG signal through multiple iterations of squaring and normalization, emphasizing its peaks. Following this, the signal is subjected to a rolling mean over a 0.75-s duration. A sliding window approach then segments the signal, with each window’s size being the product of the window duration and the sampling rate. For acceptable peak detection, constraints are set with a beats per minute (BPM) range of 40 to 180, and peak-to-peak (PP) intervals were of particular focus. A PP range is established around the mean PP interval, using either a fixed 300 milliseconds or 30% of this mean to define the upper and lower thresholds. These thresholds are crucial for discerning acceptable PP intervals, facilitating the identification of significant peaks. Furthermore, signal segments with more than three unreliable detections within 10 beats are discarded to ensure the reliability of the detected peaks.
5. Multi-Scale Peak and Trough Detection (MSPTD) by [49]	This algorithm operates by segmenting the PPG signal into overlapping windows, each spanning 6 s with a 20% overlap. Within each window, the algorithm employs the modified AMPD algorithm. This algorithm initiates by detrending the signal and computing local maxima and minima scalograms. These scalograms are matrices indicating the presence of local maxima and minima at varying scales. The method then determines the scales with the most local maxima and minima and truncates the scalograms accordingly. Peaks and onsets are identified based on these processed scalograms. After this pulse peak and pulse onset detection, the algorithm refines the peak and onset indices by searching within a 5% tolerance of the sampling frequency around the detected positions to pinpoint the exact maxima (for peaks) or minima (for onsets). After processing all windows, the detected peaks and onsets are ordered chronologically, with redundant detections discarded to ensure a unique set of pulse events.
6. Adapted Onset Detector (qppgfast) by [50]	The algorithm employs a slope sampling approach over a defined window size of 170 ms to compute the signal’s slope. For peak identification, dynamic thresholds are set. One threshold is adjusted based on a running peak value observed in the current processing interval, with this peak value being incremented by one-tenth of its difference from the threshold. A secondary threshold is established as one-third of the primary threshold. After a peak is detected, a specific lockout interval (340 ms) is applied, preventing the detection of subsequent beats for a set duration. Additionally, if no pulse was detected over an extended period, the primary threshold is reduced, provided it exceeds a minimum limit, to capture potential low-amplitude beats.
7. Symmetric Projection Attractor (SPAR) by [51,52]	This algorithm first segments the PPG data into windows, each spanning 20 s. Within each window, the average cycle length is derived using autocorrelation, due to the periodic nature of the PPG signal. This technique is bound by an HR range of 40 to 200 BPM, ensuring that the detected cycle lengths were physiologically plausible. The derived average cycle length subsequently informs the time delay parameter, which is integral to the symmetric projection attractor reconstruction (SPAR) method. This method maps the signal into two values, based on delay coordinates and specific mathematical projections. After a rotation using an optimal angle, beats are detected by pinpointing crossings of a particular line in the rotated coordinates. To ensure thorough beat detection, the algorithm adjusted for potential mismatches between windows and incorporated mechanisms to handle missed or extra beats.

**Table 3:** Demographic characteristics of 25 study participants collected at screening.

Demographic Characteristics	Total Participants (n = 25) (Mean ± Standard Deviation)
M/F (n)	8/17
Age (years)	46.92 ± 16.61
Height (cm)	168.72 ± 10.07
Weight (kg)	70.54 ± 12.40
BMI (kg/m^2^)	24.68 ± 3.10
Race	White (n = 24) American Indian or Alaska Native (n = 1)

**Table 4:** Raw data coverage from all devices—where min, max, and median values are based on the coverage across all subjects.

Day Timings	Vital Patch Median [min, max]		Corsano Median [min, max]		Whoop Median [min, max]
	Raw ECG	R-R	Raw PPG	R-R	R-R
Full Day	100 [1, 100]	98 [0, 100]	100 [33, 100]	52 [0, 94]	44 [0, 79]
Midnight to 8 a.m.	100 [1, 100]	100 [0, 100]	100 [33, 100]	88 [0, 100]	77 [0, 100]
8 a.m. to 8 p.m.	100 [1, 100]	98 [0, 100]	100 [39, 100]	31 [0, 93]	19 [0, 68]
8 p.m. to midnight	100 [3, 100]	100 [0, 100]	100 [33, 100]	75 [0, 100]	68 [0, 100]

**Table 5:** Performance of PPG-based devices compared to ECG for HR and HRV features. Accuracy between PPG- and ECG-derived measurements was quantified with mean absolute error. Agreement between PPG- and ECG-derived measurements was quantified with the spearman correlation coefficient, ICC coefficient, and Bland–Altman analysis. Bold text for each feature indicates the best performing device for the specific part of the day.

Time	Device	Mean Absolute Error	Spearman Correlation ρ (*p*-Value)	ICC (*p*-Value)	Mean Error (Bias)	Bland–Altman Limits of Agreement CI 95% (+, −)
Heart Rate, BPM						
Full Day	Corsano	1.36	0.98 (<0.001)	0.97 (<0.001)	1.14	[5.99, −3.71]
	Whoop	1.50	0.96 (<0.001)	0.93 (<0.001)	0.80	[7.47, −5.86]
Awake	Corsano	1.84	0.96 (<0.001)	0.95 (<0.001)	1.59	[7.17, −3.98]
	Whoop	1.71	0.95 (<0.001)	0.94 (<0.001)	0.90	[7.37, −5.58]
Asleep	**Corsano**	**0.85**	**0.98 (<0.001)**	**0.98 (<0.001)**	**0.65**	**[4.2, −2.9]**
	Whoop	1.31	0.96 (<0.001)	0.92 (<0.001)	0.71	[7.38, −5.96]
**Variability in Heart Rate (SD of HR), BPM**						
Full Day	Corsano	1.65	0.73 (<0.001)	0.44 (<0.001)	0.93	[7.03, −5.18]
	Whoop	1.92	0.65 (<0.001)	0.35 (<0.001)	0.68	[7.74, −6.38]
Awake	Corsano	1.98	0.56 (<0.001)	0.31 (<0.001)	0.80	[7.6, −6]
	Whoop	2.12	0.54 (<0.001)	0.33 (<0.001)	0.16	[7.42, −7.1]
Asleep	**Corsano**	**1.27**	**0.87 (<0.001)**	**0.57 (<0.001)**	**1.04**	**[6.02, −3.95]**
	Whoop	1.72	0.75 (<0.001)	0.38 (<0.001)	1.13	[7.74, −5.48]
**N−N Intervals, ms (Mean of the N-N intervals)**						
Full Day	Corsano	13.20	0.98 (<0.001)	0.98 (<0.001)	−10.38	[32.33, −53.08]
	Whoop	16.04	0.97 (<0.001)	0.96 (<0.001)	−9.49	[54.94, −73.92]
Awake	Corsano	17.10	0.97 (<0.001)	0.97 (<0.001)	−14.70	[31.88, −61.28]
	Whoop	17.93	0.96 (<0.001)	0.96 (<0.001)	−10.74	[52.78, −74.26]
Asleep	**Corsano**	**9.04**	**0.99 (<0.001)**	**0.99 (<0.001)**	**−5.83**	**[29.6, −41.26]**
	Whoop	14.28	0.97 (<0.001)	0.96 (<0.001)	−8.33	[56.12, −72.79]
**SDNN, ms (SD of the N−N intervals)**						
Full Day	Corsano	13.89	0.78 (<0.001)	0.69 (<0.001)	4.41	[50.67, −41.85]
	Whoop	17.31	0.72 (<0.001)	0.57 (<0.001)	3.18	[64.11, −57.75]
Awake	Corsano	16.47	0.62 (<0.001)	0.55 (<0.001)	1.08	[52.28, −50.12]
	Whoop	19.71	0.62 (<0.001)	0.48 (<0.001)	−3.01	[62.55, −68.57]
Asleep	**Corsano**	**10.92**	**0.91 (<0.001)**	**0.8 (<0.001)**	**7.91**	**[45.7, −29.89]**
	Whoop	15.06	0.82 (<0.001)	0.66 (<0.001)	8.79	[62.11, −44.53]
**SDSD, ms (SD of successive differences between adjacent N-N intervals)**						
Full Day	Corsano	12.52	0.7 (<0.001)	0.65 (<0.001)	−9.43	[22.77, −41.64]
	Whoop	9.42	0.76 (<0.001)	0.75 (<0.001)	−2.96	[26.07, −31.99]
Awake	Corsano	17.99	0.58 (<0.001)	0.43 (<0.001)	−15.43	[20.04, −50.9]
	Whoop	11.86	0.67 (<0.001)	0.63 (<0.001)	−6.45	[24.4, −37.3]
Asleep	**Corsano**	**6.89**	**0.89 (<0.001)**	**0.87 (<0.001)**	**−3.57**	**[18.27, −25.4]**
	Whoop	7.14	0.86 (<0.001)	0.84 (<0.001)	0.13	[25.46, −25.19]
**RMSSD, ms (Square root of mean of the sum of squares of differences between adjacent N-N intervals)**						
Full Day	Corsano	12.53	0.7 (<0.001)	0.65 (<0.001)	−9.43	[22.78, −41.64]
	Whoop	9.42	0.76 (<0.001)	0.75 (<0.001)	−2.96	[26.07, −31.99]
Awake	Corsano	17.99	0.58 (<0.001)	0.43 (<0.001)	−15.43	[20.04, −50.91]
	Whoop	11.86	0.67 (<0.001)	0.63 (<0.001)	−6.45	[24.4, −37.3]
Asleep	**Corsano**	**6.89**	**0.89 (<0.001)**	**0.87 (<0.001)**	**−3.57**	**[18.27, −25.4]**
	Whoop	7.14	0.86 (<0.001)	0.84 (<0.001)	0.13	[25.46, −25.19]
**CVSD (Coefficient of variation of successive differences between adjacent N-N intervals)**						
Full Day	Corsano	0.01	0.66 (<0.001)	0.53 (<0.001)	−0.01	[0.03, −0.05]
	Whoop	0.01	0.71 (<0.001)	0.66 (<0.001)	0.00	[0.03, −0.04]
Awake	Corsano	0.02	0.49 (<0.001)	0.32 (<0.001)	−0.02	[0.03, −0.06]
	Whoop	0.01	0.6 (<0.001)	0.54 (<0.001)	−0.01	[0.03, −0.05]
Asleep	**Corsano**	**0.01**	**0.87 (<0.001)**	**0.82 (<0.001)**	**0.00**	**[0.02, −0.03]**
	Whoop	0.01	0.82 (<0.001)	0.76 (<0.001)	0.00	[0.03, −0.03]
**CVNN (Coefficient of variation equal to the ratio of SDNN divided by Mean N-N intervals)**						
Full Day	Corsano	0.02	0.74 (<0.001)	0.59 (<0.001)	0.01	[0.06, −0.05]
	Whoop	0.02	0.68 (<0.001)	0.48 (<0.001)	0.00	[0.08, −0.07]
Awake	Corsano	0.02	0.56 (<0.001)	0.42 (<0.001)	0.00	[0.07, −0.06]
	Whoop	0.02	0.57 (<0.001)	0.4 (<0.001)	0.00	[0.08, −0.08]
Asleep	**Corsano**	**0.01**	**0.9 (<0.001)**	**0.75 (<0.001)**	**0.01**	**[0.05, −0.03]**
	Whoop	0.02	0.8 (<0.001)	0.57 (<0.001)	0.01	[0.07, −0.05]
**LF: variance (power) in HRV in the low Frequency (0.04 to 0.15 Hz), ms^2^**						
Full Day	Corsano	392.70	0.76 (<0.001)	0.45 (<0.001)	21.02	[1906.22, −1864.18]
	Whoop	427.39	0.7 (<0.001)	0.33 (<0.001)	119.11	[2195.07, −1956.84]
Awake	Corsano	479.81	0.62 (<0.001)	0.34 (<0.001)	−41.58	[2002.9, −2086.06]
	Whoop	464.56	0.61 (<0.001)	0.32 (<0.001)	44.94	[2069.97, −1980.09]
Asleep	**Corsano**	**282.50**	**0.89 (<0.001)**	**0.61 (<0.001)**	**75.75**	**[1627.54, −1476.04]**
	Whoop	386.94	0.78 (<0.001)	0.35 (<0.001)	182.01	[2218.12, −1854.1]
**HF: variance (power) in HRV in the High Frequency (0.15 to 0.40 Hz), ms^2^**						
Full Day	Corsano	312.58	0.66 (<0.001)	0.29 (<0.001)	−49.69	[1519.79, −1619.16]
	Whoop	268.87	0.68 (<0.001)	0.25 (<0.001)	63.74	[1769.18, −1641.71]
Awake	Corsano	404.71	0.56 (<0.001)	0.21 (<0.001)	−142.96	[1525.31, −1811.23]
	Whoop	298.56	0.6 (<0.001)	0.22 (<0.001)	4.81	[1591.43, −1581.81]
Asleep	**Corsano**	**202.24**	**0.84 (<0.001)**	**0.43 (<0.001)**	**34.86**	**[1351.93, −1282.2]**
	Whoop	236.42	0.78 (<0.001)	0.28 (<0.001)	112.07	[1845.99, −1621.85]
**Ratio: LF/HF**						
Full Day	Corsano	1.62	0.65 (<0.001)	0.51 (<0.001)	1.24	[6.17, −3.7]
	Whoop	1.34	0.69 (<0.001)	0.59 (<0.001)	0.80	[5.24, −3.65]
Awake	Corsano	2.16	0.55 (<0.001)	0.29 (<0.001)	1.93	[7.6, −3.74]
	Whoop	1.58	0.57 (<0.001)	0.44 (<0.001)	1.15	[6.03, −3.72]
Asleep	**Corsano**	**1.13**	**0.8 (<0.001)**	**0.73 (<0.001)**	**0.60**	**[4.46, −3.26]**
	Whoop	1.17	0.78 (<0.001)	0.69 (<0.001)	0.51	[4.57, −3.55]
**Sample Entropy**						
Full Day	Corsano	0.41	0.45 (<0.001)	0.4 (<0.001)	−0.23	[0.69, −1.15]
	Whoop	0.45	0.45 (<0.001)	0.4 (<0.001)	0.21	[1.3, −0.87]
Awake	Corsano	0.46	0.32 (<0.001)	0.26 (<0.001)	−0.30	[0.7, −1.29]
	Whoop	0.59	0.32 (<0.001)	0.25 (<0.001)	0.41	[1.62, −0.8]
Asleep	Corsano	0.36	0.58 (<0.001)	0.53 (<0.001)	−0.17	[0.67, −1.01]
	**Whoop**	**0.33**	**0.65 (<0.001)**	**0.61 (<0.001)**	**0.05**	**[0.88, −0.79]**

**Table 6:** Reliability assessment of HR and HRV features in daily life. Values in the table indicate the magnitude of intraclass correlation between Day 1 and Day 2 and their corresponding *p*-values in parenthesis.

Feature	Timings of the Day					
	Full Day		Awake		Asleep	
	Dominant	Non-Dominant	Dominant	Non-Dominant	Dominant	Non-Dominant
Heart Rate	0.72 (<0.001)	0.66 (<0.001)	0.65 (<0.001)	0.47 (<0.001)	0.68 (<0.001)	0.66 (<0.001)
SD HR	0.26 (<0.001)	0.33 (<0.001)	0.25 (<0.001)	0.20 (<0.001)	0.33 (<0.001)	0.35 (<0.001)
Mean N-N	0.76 (<0.001)	0.70 (<0.001)	0.71 (<0.001)	0.55 (<0.001)	0.67 (<0.001)	0.69 (<0.001)
SDNN	0.44 (<0.001)	0.42 (<0.001)	0.31 (<0.001)	0.26 (<0.001)	0.47 (<0.001)	0.42 (<0.001)
SDSD	0.70 (<0.001)	0.61 (<0.001)	0.30 (<0.001)	0.38 (<0.001)	0.77 (<0.001)	0.68 (<0.001)
RMSSD	0.70 (<0.001)	0.61 (<0.001)	0.30 (<0.001)	0.38 (<0.001)	0.77 (<0.001)	0.68 (<0.001)
CVSD	0.63 (<0.001)	0.59 (<0.001)	0.21 (<0.001)	0.31 (<0.001)	0.73 (<0.001)	0.66 (<0.001)
CVNN	0.32 (<0.001)	0.35 (<0.001)	0.20 (<0.001)	0.19 (<0.001)	0.41 (<0.001)	0.41 (<0.001)
LF	0.33 (<0.001)	0.31 (<0.001)	0.22 (<0.001)	0.09 (0.015)	0.34 (<0.001)	0.27 (<0.001)
HF	0.62 (<0.001)	0.59 (<0.001)	0.40 (<0.001)	0.33 (<0.001)	0.65 (<0.001)	0.60 (<0.001)
Ratio: LF/HF	0.39 (<0.001)	0.30 (<0.001)	0.69 (<0.001)	0.50 (<0.001)	0.28 (<0.001)	0.20 (<0.001)
Sample Entropy	0.11 (<0.001)	0.09 (<0.001)	0.05 (0.151)	0.08 (0.035)	0.20 (<0.001)	0.09 (0.001)

**Table 7:** Performance metric of PPG beat detectors compared to R-peak detected from ECG in daily life recordings. All the performance metrics are reported as median values along with 95% confidence intervals.

Task	Metric	Beat Detector Selection on the Full Day PPG Data						
		MSPTD	AMPD	ERMA	ABD	SPAR	HeartPy	qppgfast
Beat Detection	F1 Score	62.54 [45.10, 86.95]	62.18 [44.70, 86.89]	61.81 [44.68, 86.61]	61.49 [44.32, 86.28]	61.03 [44.13, 86.37]	60.58 [42.29, 86.60]	59.48 [43.05, 84.78]
	Sensitivity	61.66 [44.48, 94.78]	60.67 [43.60, 89.90]	59.85 [42.91, 86.55]	59.93 [43.07, 86.03]	58.92 [42.51, 85.70]	55.68 [37.23, 84.74]	57.61 [38.82, 84.35]
	PPV	63.03 [44.33, 89.79]	63.83 [44.80, 90.02]	64.16 [45.14, 89.29]	63.56 [44.80, 89.19]	64.11 [45.09, 88.79]	66.21 [45.74, 90.51]	61.35 [44.66, 85.20]
HR Estimation, BPM	MAPE	9.31 [1.18, 19.26]	9.89 [1.27, 20.04]	9.49 [1.26, 18.48]	11.06 [1.38, 19.35]	11.12 [3.31, 20.03]	14.85 [1.73, 27.06]	14.89 [6.87, 38.95]
	MAE	7.27 [2.31, 15.87]	7.59 [2.33, 18.02]	7.24 [2.38, 16.11]	8.44 [2.99, 17.74]	8.89 [2.86, 19.29]	11.28 [3.51, 23.48]	11.57 [4.83, 25.78]
	Bias	−1.91 [−9.83, 3.58]	−3.58 [−12.96, 1.00]	−3.46 [−14.35, 1.48]	−3.78 [−13.00, 2.24]	−5.63 [−16.08, −0.79]	−8.61 [−20.71, −0.78]	−1.91 [−11.15, 15.63]
	LOA	[12.29, 57.37]	[12.27, 52.21]	[11.96, 38.47]	[13.89, 42.02]	[12.55, 44.79]	[16.68, 50.11]	[19.77, 60.13]
Inter Beat Interval, ms	MAE	249.68 [118.52, 439.18]	269.88 [133.50, 461.53]	337.57 [139.99, 895.76]	355.39 [182.3, 644.1]	309.57 [174.3, 516.9]	637.15 [286.8, 2143.03]	951.21 [259.5, 2949.68]
	Bias	−54.27 [−249.82, 61.42]	−23.79 [−232.97, 97.49]	25.84 [−168.32, 651.07]	42.92 [−229.3, 342.66]	21.54 [−207.3, 165.29]	328.71 [−23.4, 1938.50]	564.21 [−19.66, 2621.57]
	LOA	[620.64, 3228.51]	[664.93, 3276.37]	[792.02, 10956.50]	[844.73, 7507.64]	[815.26, 3363.01]	[1413.30, 13,584.81]	[2692.00, 27,274.76]

**Table 8:** Top performing PPG beat detector (MSPTD) performance under awake and asleep periods. All the performance metrics are reported as median values along with 95% confidence intervals.

Task	Metric	MSPTD Performance during Awake and Asleep Period	
		Awake	Asleep
Beat Detection	F1 Score	55.90 [43.26, 83.75]	89.25 [50.91, 99.37]
	Sensitivity	54.76 [43.79, 86.48]	89.94 [50.85, 99.46]
	PPV	57.62 [41.18, 86.27]	89.45 [50.98, 99.34]
HR Estimation, BPM	MAPE	12.58 [3.17, 22.91]	1.78 [0.65, 18.60]
	MAE	10.01 [3.67, 20.84]	1.12 [0.41, 14.47]
	Bias	−3.44 [−15.47, 3.29]	0.03 [−4.39, 7.66]
	LOA	[14.64, 59.31]	[2.44, 75.21]
Inter Beat Interval, ms	MAE	219.99 [107.23, 421.95]	53.80 [25.94, 205.58]
	Bias	67.29 [−243.54, 189.07]	−4.50 [−58.29, 65.19]
	LOA	[450.25, 2534.76]	[129.31, 1457.40]

**Figure A1:**
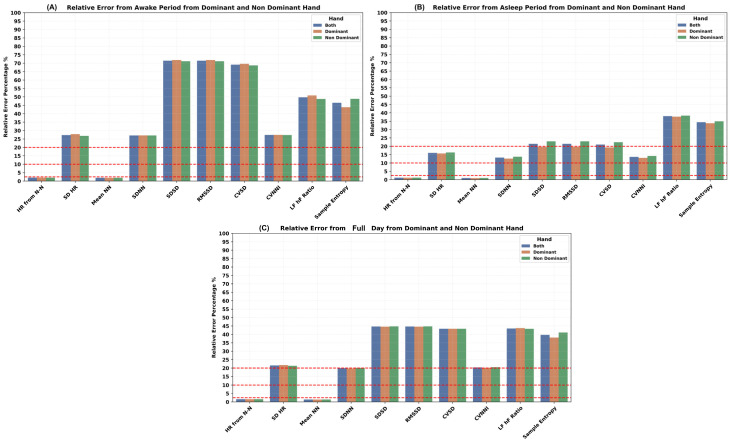
Impact of dominant vs. non-dominant wrist on accuracy of PPG-derived HR and HRV features.

## Data Availability

The data sharing policy of Janssen Pharmaceutical Companies of Johnson and Johnson is available at https://www.janssen.com/clinical-trials/transparency (accessed 21 October 2024). Requests for access to the study data can be submitted through Yale Open Data Access (YODA) project site at http://yoda.yale.edu (accessed 21 October 2024).

## Appendix A. Impact of Dominant vs. Non-Dominant Hand on PPG Device Performance

Impact of the dominant vs. non-dominant hand wearing position on the accuracy of PPG derived HR/HRV features was explored during awake (Figure A1A), asleep (Figure A1B), and full day periods (Figure A1C). Overall, for all the HR/HRV features, the difference between the dominant and non-dominant hands was not significant. During the awake period, the dominant hand had slightly higher error for time domain HRV compared to the non-dominant hand. However, during the asleep period, the non-dominant hand had a slightly higher error compared to the dominant hand for all HRV features. During the full day period, the difference between the dominant and non-dominant hand was negligible.

## Appendix B. Impact of Interpolation and No-Interpolation Within the 5-min Epoch of N-N Intervals on the Accuracy of the HRV Features (e.g., SDNN, RMSSD)

This topic of signal preprocessing and its impact on heart rate variability (HRV) metrics has already been investigated in greater detail, particularly in a study such as “Effects of Missing Data on Heart Rate Variability Metrics” by [41]. According to this paper, “*The optimal correction methodology for HRV metrics varies: correction without gap filling is superior for SDNN, RMSSD, and Poincaré plot metrics when missing beats occur predominantly in bursts, while gap-filling methods are advantageous for instances of sporadic missing beats. Gap-filling methodologies achieved optimal performance regarding frequency-domain parameters”.* There are mixed results in terms of which features to interpolate. This study [41] highlights that different interpolation techniques (including linear interpolation) have varying effects on HRV metrics, depending on the nature of the missing data. For example, while linear interpolation works well for scattered missing beats, other approaches may be more suitable for burst-type data loss. However, to simplify the preprocessing for our validation study, we opted to use linear interpolation for all HRV features rather than adopting different interpolation strategies for each feature. We prioritized the practical application in real-world settings, allowing for typical interruptions like motion artifacts or signal dropouts, with a 40% data presence threshold per epoch to ensure robustness. Additionally, an ablation study comparing SDNN and RMSSD metrics, with and without interpolation, showed improved results with longer epoch coverage, as seen in Table A1.

**Table A1:** Impact of interpolation and no-interpolation on the performance of the HRV features (e.g., SDNN, RMSSD) when extracted from a PPG-based device and compared with an ECG-based device.

Time	Interpolation/ No-Interpolation	Mean Absolute Error	Spearman Correlation ρ (*p*-Value)	ICC (*p*-Value)	Mean Error (Bias)	Bland–Altman Limits of Agreement CI 95% (+, −)
**SDNN, ms (SD of the N-N intervals)**						
Full Day	Interpolation	13.89	0.78 (<0.001)	0.69 (<0.001)	4.41	[50.67, −41.85]
	No-Interpolation	12.67	0.80 (<0.001)	0.72 (<0.001)	4.36	[46.60, −37.87]
Awake	Interpolation	16.47	0.62 (<0.001)	0.55 (<0.001)	1.08	[52.28, −50.12]
	No-Interpolation	14.92	0.66 (<0.001)	0.59 (<0.001)	1.53	[48.22, −45.15]
Asleep	Interpolation	10.92	0.91 (<0.001)	0.80 (<0.001)	7.91	[45.7, −29.89]
	No-Interpolation	10.09	0.92 (<0.001)	0.82 (<0.001)	7.34	[42.07, −27.37]
**RMSSD, ms (Square root of mean of the sum of squares of differences between adjacent N-N intervals)**						
Full Day	Interpolation	12.53	0.7 (<0.001)	0.65 (<0.001)	−9.43	[22.78, −41.64]
	No-Interpolation	16.76	0.65 (<0.001)	0.53 (<0.001)	−10.97	[38.31, −60.25]
Awake	Interpolation	17.99	0.58 (<0.001)	0.43 (<0.001)	−15.43	[20.04, −50.91]
	No-Interpolation	23.98	0.50 (<0.001)	0.32 (<0.001)	−18.92	[35.92, −73.75]
Asleep	Interpolation	6.89	0.89 (<0.001)	0.87 (<0.001)	−3.57	[18.27, −25.4]
	No-Interpolation	9.13	0.85 (<0.001)	0.79 (<0.001)	−3.16	[30.86, −37.18]

The ablation study in Table A1 shows that for SDNN, no-interpolation slightly improves performance, especially during awake periods, while RMSSD performs better with interpolation, particularly when handling larger data gaps. These findings confirm our methodology’s robustness, suggesting that no-interpolation may be beneficial for SDNN, but linear interpolation helps manage RMSSD variability due to missing data.
